# Sex‐Specific Associations With Abnormal Myocardial Flow Reserve in Non‐Obstructive Coronary Artery Disease: Insights From a Real‐World Cadmium‐Zinc‐Telluride SPECT Study

**DOI:** 10.1002/clc.70294

**Published:** 2026-04-23

**Authors:** Ya‐Jie Wang, Jian‐Ming Li, Xue Wu, Tong Liu, Kang‐Yin Chen

**Affiliations:** ^1^ Tianjin Key Laboratory of Ionic‐Molecular Function of Cardiovascular Disease, Department of Cardiology Tianjin Institute of Cardiology, the Second Hospital of Tianjin Medical University Tianjin China; ^2^ Department of Cardiology TEDA International Cardiovascular Hospital, Clinical Cardiovascular Institute of Tianjin Medical University Tianjin China; ^3^ Department of Nuclear Medicine TEDA International Cardiovascular Hospital, Clinical Cardiovascular Institute of Tianjin Medical University Tianjin China; ^4^ Institute for Global Health Sciences University of California San Francisco California USA

**Keywords:** cadmium‐zinc‐telluride SPECT, coronary microvascular dysfunction, myocardial flow reserve, non‐obstructive coronary artery disease, sex differences

## Abstract

**Background:**

Coronary microvascular dysfunction (CMVD) is prevalent in non‐obstructive coronary artery disease (NOCAD), and potential sex‐related determinants remain incompletely characterized. This study explored sex‐specific associations with abnormal myocardial flow reserve (MFR) using cadmium‐zinc‐telluride single‐photon emission computed tomography (CZT‐SPECT) in a real‐world cohort.

**Methods:**

We retrospectively analyzed 393 NOCAD patients (56.7% female) undergoing CZT‐SPECT. Abnormal MFR was defined as < 2.5. Candidate variables were identified through group comparisons (*p* < 0.10) and univariate logistic regression, followed by multicollinearity assessment. A pooled multivariable logistic regression model incorporating main effects and sex interaction terms was constructed. Receiver operating characteristic (ROC) analysis evaluated discriminatory performance.

**Results:**

Abnormal MFR was observed in 49.1% of patients. In the multivariable model, LDL‐C was associated with abnormal MFR (OR: 1.471, 95% CI: 1.072–2.018; *p* = 0.017). A significant sex × MDRD‐eGFR interaction was identified (OR: 1.024, 95% CI: 1.003–1.046; *p* = 0.026), with a borderline sex × LDL‐C interaction (OR: 0.650; *p* = 0.089). The overall model demonstrated moderate discrimination (AUC: 0.680). In females, LDL‐C showed modest discriminatory ability (AUC: 0.586). Among patients with abnormal MFR, females exhibited higher LCX and RCA territory resting myocardial blood flow and higher E/e', and RCA‐rMBF was inversely correlated with E/e'.

**Conclusions:**

Sex‐modified associations of renal and lipid parameters with abnormal MFR were observed in this cohort. These interaction patterns and female‐specific phenotypes provide preliminary insights into cardiorenal‐microvascular relationships in CMVD, but should be interpreted as exploratory and hypothesis‐generating and warrant further validation.

## Graphical abstract

In 393 NOCAD patients undergoing CZT‐SPECT, LDL‐C was independently associated with abnormal MFR, with a significant sex×MDRD‐eGFR interaction. Female patients showed modest LDL‐C discrimination and distinct perfusion–diastolic phenotypes, suggesting sex‐modified cardiorenal‐microvascular relationships in coronary microvascular dysfunction.

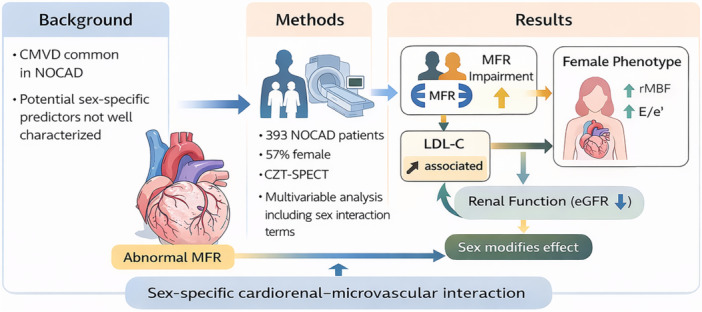

**Abbreviations**
AUC: Area Under the Curve
BMI: Body Mass Index
CAD: Coronary Artery Disease
CAG: Coronary Angiography
CI: Confidence Interval
CMVD: Coronary Microvascular Dysfunction
Cr: Creatinine
CT: Computed Tomography
CTA: Coronary Computed Tomography Angiography
CZT‐SPECT: Cadmium‐Zinc‐Telluride Single‐Photon Emission Computed Tomography
DBP: Diastolic Blood Pressure
D‐MPI: Dynamic Myocardial Perfusion Imaging
eGFR: Estimated Glomerular Filtration Rate
E/e′: Ratio of early diastolic transmitral flow velocity (E) to early diastolic mitral annular tissue velocity (e′)
FBG: Fasting Blood Glucose
Hb: Hemoglobin
HDL‐C: High‐Density Lipoprotein Cholesterol
HR: Heart Rate
IVS: Interventricular Septal Thickness
LA: Left Atrial Diameter
LAD‐MFR: Left Anterior Descending Myocardial Flow Reserve
LAD‐rMBF: LAD Resting Myocardial Blood Flow
LAD‐sMBF: LAD Stress Myocardial Blood Flow
LCX: Left Circumflex Coronary Artery
LCX‐MFR: Left Circumflex Myocardial Flow Reserve
LCX‐rMBF: LCX Resting Myocardial Blood Flow
LCX‐sMBF: LCX Stress Myocardial Blood Flow
LDL‐C: Low‐Density Lipoprotein Cholesterol
LV: Left Ventricle
LVEDD: Left Ventricular End‐Diastolic Diameter
LVEF: Left Ventricular Ejection Fraction
LVPW: Left Ventricular Posterior Wall Thickness
LV‐EDV: Left Ventricular End‐Diastolic Volume
LV‐ESV: Left Ventricular End‐Systolic Volume
LV‐MFR: Left Ventricular Myocardial Flow Reserve
LV‐rMBF: Left Ventricular Resting Myocardial Blood Flow
LV‐sMBF: Left Ventricular Stress Myocardial Blood Flow
MBF: Myocardial Blood Flow
MBq: Megabecquerel
MDRD‐eGFR: Estimated Glomerular Filtration Rate by the Modification of Diet in Renal Disease (MDRD) equation
MFR: Myocardial Flow Reserve
NOCAD: Non‐Obstructive Coronary Artery Disease
NYHA: New York Heart Association
OR: Odds Ratio
PET/CT: Positron Emission Tomography/Computed Tomography
PLT: Platelet Count
RA: Right Atrial Diameter
RCA: Right Coronary Artery
RCA‐MFR: Right Coronary Artery Myocardial Flow Reserve
RCA‐rMBF: RCA Resting Myocardial Blood Flow
RCA‐sMBF: RCA Stress Myocardial Blood Flow
rMBF: Resting Myocardial Blood Flow
ROC: Receiver Operating Characteristic
RV: Right Ventricular Diameter
SBP: Systolic Blood Pressure
sMBF: Stress Myocardial Blood Flow
SPECT: Single‐Photon Emission Computed Tomography
SV: Stroke Volume
TC: Total Cholesterol
TG: Triglycerides
TyG: Triglyceride‐Glucose Index
UA: Uric Acid
VIF: Variance Inflation Factor
WBC: White Blood Cell Count

## 1 Introduction

Since Likoff et al. first reported patients with angina and normal coronary angiography (CAG) in 1967, research on coronary microvascular disease (CMVD) has advanced over the past half‐century, resulting in numerous international consensus documents that systematically review its progress [1, 2, 3, 4, 5]. In clinical practice, evaluating coronary microvascular function in patients with non‐obstructive coronary artery disease (NOCAD) to aid in diagnosing CMVD has gained increasing attention. This assessment is critical, as it enhances risk stratification, improves prognostic accuracy, and supports quality of life by guiding tailored therapeutic interventions [6, 7, 8, 9].

Among functional assessments for CMVD, non‐invasive methods are favored over invasive techniques due to their safety, patient comfort, wide applicability, and cost‐effectiveness. Positron Emission Tomography/Computed Tomography (PET/CT) is regarded as the gold standard for these evaluations [10]. More recently, Cadmium‐Zinc‐Telluride Single‐Photon Emission Computed Tomography (CZT‐SPECT) has emerged as a promising alternative [11, 12, 13]. With enhanced detector technology and imaging algorithms, CZT‐SPECT demonstrates concordance with PET/CT in detecting myocardial ischemia and quantifying myocardial blood flow (MBF). Furthermore, its cost‐effectiveness and access to radioisotopes suggest broader clinical adoption in the future [14].

Previous studies have identified significant sex differences in the epidemiology, symptomatology, pathophysiology, imaging findings, and prognosis of CMVD [15, 16, 17, 18, 19]. These findings underscore the necessity for sex‐specific diagnostic and therapeutic strategies to optimize patient outcomes [20]. The objective of this study is to investigate sex‐specific associations with abnormal myocardial flow reserve (MFR) in NOCAD patients using CZT‐SPECT in a real‐world cohort.

## 2 Materials and Methods

### 2.1 Patients Selection and Baseline Information Collection

Hospitalizations occurring between March 1, 2021 and March 31, 2022 in the Department of Internal Medicine (Division I) were retrospectively screened. Exclusion criteria included: (1) Prior confirmed CAD, including previous CAG confirming stenosis ≥ 50%, coronary intervention, bypass surgery, or myocardial infarction. Patients without a clear myocardial infarction history but with evidence on current CZT‐SPECT imaging were also excluded; (2) Patients with ≥ 50% stenosis in any coronary artery segment or ≥ 50% systolic compression from a myocardial bridge on invasive CAG. Those with moderate or severe stenosis or a myocardial bridge on Computed Tomography (CT) angiography were also excluded; (3) Structural heart diseases such as congenital heart disease, valvular disease, cardiomyopathy, or heart failure with a left ventricular ejection fraction (LVEF) < 50% (NYHA class II–IV); (4) Significant coronary anatomical variations, such as ectopic coronary origins, coronary fistula, or single coronary anomalies; (5) Severe arrhythmias, including sinus arrest, atrioventricular block, or sustained ventricular or supraventricular tachycardia; (6) Other severe medical conditions, including chronic obstructive pulmonary disease, asthma, severe allergic reactions, severe renal insufficiency, or malignancies.

Medical records were reviewed to collect basic patient information, including sex, age, height, weight, and medical history (such as CAD, hypertension, diabetes, and smoking status). Height and weight were measured on admission, with body mass index (BMI) calculated accordingly. Hypertension was defined as a prior diagnosis or a new diagnosis based on continuous blood pressure monitoring during the current hospitalization. Similarly, diabetes was defined as a prior diagnosis or new diagnosis based on laboratory tests and blood glucose monitoring. Current smoking was defined as regular smoking or having quit less than 1 year before admission. This retrospective study was approved by the Ethics Committee of TEDA International Cardiovascular Hospital (Approval No. [2022]‐0720‐2), and the requirement for individual informed consent was waived due to the retrospective design, use of anonymized routine clinical records, and general consent for research purposes obtained upon hospitalization.

### 2.2 Patient Identification and Selection

During the study period (March 1, 2021 to March 31, 2022), a total of 1560 hospitalizations occurred in the Department of Internal Medicine (Division I). These records encompassed all admissions during the study period regardless of primary diagnosis. Subsequently, initial case identification was performed using hospital discharge diagnostic codes related to suspected ischemic heart disease, yielding 1113 hospitalization records. Procedural records were subsequently reviewed to exclude hospitalizations involving percutaneous coronary intervention during the index admission. After this step, 785 hospitalization records remained for detailed evaluation. All 785 records underwent individual manual chart review to confirm eligibility according to predefined inclusion and exclusion criteria. Coronary imaging findings (invasive CAG or CTA) were reviewed to exclude obstructive epicardial coronary artery disease according to the prespecified thresholds detailed in the exclusion criteria. Other exclusion criteria (e.g., prior coronary revascularization, myocardial infarction, structural heart disease, significant coronary anatomical anomalies, severe arrhythmias, and other severe medical conditions) were confirmed through comprehensive review of clinical history and relevant diagnostic records. Availability of CZT‐SPECT imaging and completeness of laboratory and echocardiographic data were also confirmed. During manual review, duplicate admissions were identified and consolidated to ensure that each included case corresponded to a unique individual. A total of 393 unique patients met all criteria and were included in the final analysis (Figure S1).

### 2.3 Vital Signs Measurement

After admission, patients generally continued their pre‐hospital medications according to routine clinical practice. They were typically instructed to fast from 10:00 PM on the day of admission. On the following morning, while awake and resting supine for at least 10 min in a quiet environment, blood pressure and heart rate were measured. A trained nurse used an electronic blood pressure monitor (TERUMO Elemano), selecting the appropriately sized cuff based on each patient's arm circumference. Blood pressure was measured in both upper limbs, and the higher value recorded. Systolic blood pressure (SBP), diastolic blood pressure (DBP), and heart rate (HR) values were documented.

### 2.4 Laboratory Tests

Venous blood samples were collected in the morning following admission for routine hematologic and biochemical analyses. The recorded parameters included white blood cell (WBC) count, hemoglobin (Hb), platelet count (PLT), fasting blood glucose (FBG), creatinine (Cr), uric acid (UA), total cholesterol (TC), triglycerides (TG), high‐density lipoprotein cholesterol (HDL‐C), and low‐density lipoprotein cholesterol (LDL‐C). Based on laboratory results and individual patient data, the estimated glomerular filtration rate (eGFR) was calculated using the simplified MDRD formula, and the triglyceride‐glucose (TyG) index was determined. Information regarding baseline lipid‐lowering therapy (e.g., statins, ezetimibe, PCSK9 inhibitors) was not consistently documented in the retrospective medical records and therefore was not available for adjustment in the present analysis.

### 2.5 Coronary Imaging and Echocardiography

During hospitalization, patients underwent either invasive CAG or coronary computed tomography angiography (CTA) to assess the epicardial coronary arteries and identify NOCAD. For invasive angiography, standard protocols with at least two projection views per artery were followed. Two senior interventional cardiologists independently reviewed the results, with a third consulted if discrepancies arose, and a consensus was reached through discussion among the three. For coronary CTA, both inpatient tests and outpatient results from within 2 weeks prior to admission were accepted. The GE 256‐slice spiral CT scanner (Revolution CT) was used, with measurements processed by post‐processing workstation software. Sublingual nitroglycerin (0.5 mg) was administered 3–5 min before scanning.

During hospitalization, trained echocardiographers performed transthoracic echocardiography using standard methods. Two‐dimensional echocardiography was used to measure interventricular septal (IVS) thickness, left ventricular end‐diastolic diameter (LVEDD), left ventricular posterior wall (LVPW) thickness, left atrial (LA) diameter, right ventricular (RV) diameter, and right atrial (RA) diameter. Left ventricular end‐diastolic volume (LV‐EDV) and left ventricular end‐systolic volume (LV‐ESV) were measured using two‐dimensional echocardiography combined with the biplane Simpson's method, and stroke volume (SV) and LVEF were calculated. Pulsed Doppler was used to measure the early diastolic filling velocity (E wave) at the mitral valve in the apical four‐chamber view, while tissue Doppler imaging measured the early diastolic velocity (e’ wave) at the mitral annulus in the lateral wall and interventricular septum, with the E/e’ ratio calculated.

### 2.6 CZT‐SPECT Myocardial Perfusion Imaging

All patients underwent dynamic myocardial perfusion imaging (D‐MPI) using a CZT‐based dedicated cardiac SPECT system with technetium‐99m sestamibi (^99m^Tc‐sestamibi) as the imaging agent. Patients were instructed to avoid foods and beverages containing caffeine or theophylline for 24 h before the test, and cardiovascular medications (including nitrates, nicorandil, calcium channel blockers, and beta‐blockers) were withheld for at least 24 h prior to testing. A single‐day imaging protocol was used, with resting D‐MPI performed first with 185–296 megabecquerels (MBq) of ^99m^Tc‐sestamibi, followed by stress D‐MPI 1–4 h later. During peak adenosine stress (0.14 mg/kg/min, administered via intravenous infusion for 3 min), 555–888 MBq of ^99m^Tc‐sestamibi was injected. Dynamic and gated imaging were performed with attenuation correction by low‐dose CT. Resting myocardial blood flow (rMBF) and stress myocardial blood flow (sMBF) were measured and analyzed using a dedicated workstation, and MFR was calculated. Based on the global MFR value of the left ventricle, patients were classified into two groups: those with MFR ≥ 2.5 were considered normal (normal MFR group), while those with MFR < 2.5 were classified as abnormal (abnormal MFR group). This cutoff of 2.5 was selected based on established thresholds for detecting coronary microvascular dysfunction.

### 2.7 Statistical Analysis

Continuous variables were assessed for normality using the Kolmogorov–Smirnov test. Normally distributed variables were expressed as mean ± standard deviation, while non‐normally distributed variables were presented as median[interquartile range]. Categorical variables were reported as *n*(%). Comparisons between groups were performed using independent *t‐*tests for normally distributed continuous variables, Mann–Whitney *U* tests for non‐normally distributed continuous variables, and chi‐square tests or Fisher's exact tests for categorical variables, with statistical significance defined as *p* < 0.05 and trends as *p* < 0.1. Variables with significant differences (*p* < 0.05) or trends (*p* < 0.1) from group comparisons were selected for further analysis. Univariate logistic regression was then applied to these variables, with those exhibiting *p* < 0.1 retained for subsequent investigation. Multicollinearity among these variables was evaluated using variance inflation factor (VIF) analysis, with variables exhibiting VIF > 10 excluded to avoid parameter instability and biased estimates. A pooled multivariable logistic regression model was then employed to assess associations with abnormal MFR. The model included main effects and interaction terms to evaluate sex‐specific effects, with odds ratios (OR) and 95% confidence intervals (CI) reported. Statistical significance was defined as *p* < 0.05, with *p* < 0.1 indicating a trend. Receiver operating characteristic (ROC) curve analysis was performed to evaluate the discriminatory performance of the pooled model and selected predictors. The area under the curve (AUC) with 95% CI was calculated to assess the ability to distinguish abnormal from normal MFR. ROC curves were generated for the model's predicted probability to evaluate overall performance and for key predictors both in the overall cohort and stratified by sex. All statistical analyses were performed using SPSS Statistics for Windows, Version 31.0 (IBM Corp., Armonk, NY, USA), with a two‐sided *p*‐value threshold of 0.05 for significance unless otherwise specified.

## 3 Results

### 3.1 Patient Characteristics and Study Cohort

The study included 393 patients with NOCAD, with ages ranging from 27 to 84 years (median age 61 years). Hypertension was present in 56.0% (220/393) of patients, diabetes in 25.7% (101/393), and current smoking in 22.6% (89/393). Among females (223/393, 56.7%), 89.2% (199/223) were postmenopausal. Coronary imaging was performed using invasive CAG in 92.9% (365/393) and coronary CTA in 7.1% (28/393). Among females, CAG was conducted in 93.7% (209/223) and CTA in 6.3% (14/223); among males, CAG was used in 91.8% (156/170) and CTA in 8.2% (14/170). Abnormal MFR (MFR < 2.5) was identified in 49.1% (193/393) of patients, with no significant sex difference in prevalence (*p* = 0.609).

### 3.2 Statistical Analysis and Interaction Findings

To explore factors associated with abnormal MFR (< 2.5), group comparisons between normal (MFR ≥ 2.5) and abnormal groups were performed in the overall cohort as well as in sex‐stratified analyses. Variables demonstrating statistical significance (*p* < 0.05) or a trend toward significance (*p* < 0.10) included age, diabetes, postmenopausal status, FBG, TC, TG, LDL‐C, UA, Cr, MDRD‐eGFR, TyG index, LVEDD, LA, LVPW, and E/e' ratio (Table 1 and Table S1). In sex‐stratified comparisons, females with abnormal MFR were older and showed a more adverse cardiometabolic and diastolic profile, including higher LDL‐C/TC, higher creatinine with lower MDRD‐eGFR, larger LA diameter, thicker LVPW, and higher E/e’ ratio (all *p* < 0.05). In contrast, in males abnormal MFR was primarily associated with a larger LVEDD (*p* = 0.014).

**Table 1:** Sex‐stratified comparison of patient characteristics by MFR status.

	Female NOCAD patients			Male NOCAD patients		
	Normal MFR group (*n* = 116)	Abnormal MFR group (*n* = 107)	*p* value	Normal MFR group (*n* = 84)	Abnormal MFR group (*n* = 86)	*p* value
**CZT‐SPECT parameters**						
LV‐MFR	3.19 [0.85]	1.86 [0.68]	< 0.001*	3.40 ± 0.55	1.89 ± 0.41	< 0.001*
LAD‐MFR	3.15 [0.93]	1.88 [0.66]	< 0.001*	3.36 ± 0.59	1.92 ± 0.44	< 0.001*
LCX‐MFR	2.97 ± 0.61	1.69 ± 0.45	< 0.001*	3.17 [0.75]	1.82 [0.59]	< 0.001*
RCA‐MFR	3.48 ± 0.66	1.91 ± 0.48	< 0.001*	3.61 ± 0.74	1.97 ± 0.47	< 0.001*
LV‐rMBF	0.90 [0.14]	0.89 [0.12]	0.862	0.90 [0.20]	0.88 [0.14]	0.582
LV‐sMBF	2.81 ± 0.68	1.61 ± 0.41	< 0.001*	2.84 ± 0.77	1.56 ± 0.43	< 0.001*
LAD‐rMBF	0.91 [0.19]	0.90 [0.15]	0.968	0.91 [0.17]	0.91 [0.13]	0.814
LAD‐sMBF	2.71 [1.03]	1.59 [0.50]	< 0.001*	2.84 ± 0.79	1.62 ± 0.46	< 0.001*
LCX‐rMBF	0.85 [0.15]	0.85 [0.15]	0.975	0.83 [0.23]	0.82 [0.22]	0.529
LCX‐sMBF	2.51 ± 0.67	1.44 ± 0.45	< 0.001*	2.63 ± 0.70	1.39 ± 0.44	< 0.001*
RCA‐rMBF	0.91 [0.19]	0.91 [0.16]	0.704	0.89 [0.25]	0.88 [0.19]	0.494
RCA‐sMBF	3.06 [1.39]	1.59 [0.72]	< 0.001*	3.03 ± 1.05	1.63 ± 0.53	< 0.001*
**Baseline characteristics**						
Age	60.72 ± 8.48	62.99 ± 7.85	0.040*	58.50 [12.75]	61.50 [15.25]	0.322
BMI	25.02 [4.91]	24.97 [4.35]	0.983	26.40 ± 3.86	25.93 ± 3.67	0.412
Hypertension	63 (54.3%)	61 (57.0%)	0.685	49 (58.3%)	47 (54.7%)	0.628
Diabetes	29 (25.0%)	32 (29.9%)	0.412	15 (17.9%)	25 (29.1%)	0.085^†^
Current smoking	11 (9.5%)	14 (13.1%)	0.394	28 (33.3%)	36 (41.9%)	0.251
Postmenopausal	99 (85.3%)	100 (93.5%)	0.051^†^	—	—	—
**Vital Signs**						
Resting SBP	132.00 [23.00]	133.00 [19.00]	0.557	133.99 ± 14.66	134.20 ± 14.69	0.926
Resting DBP	79.00 [13.75]	79.00 [13.00]	0.965	82.00 ± 11.79	80.72 ± 10.08	0.448
Resting HR	70.50 [16.00]	72.00 [12.00]	0.582	73.50 [14.75]	70.50 [14.00]	0.172
**Laboratory values**						
WBC	5.50 [1.90]	5.30 [1.80]	0.806	5.90 [2.08]	5.70 [1.73]	0.783
Hb	131.26 ± 10.29	133.03 ± 9.60	0.187	150.92 ± 16.07	150.65 ± 14.08	0.909
PLT	247.27 ± 66.13	247.20 ± 55.89	0.993	220.00 [54.50]	210.00 [76.50]	0.282
FBG	5.30 [1.40]	5.40 [1.70]	0.240	5.20 [1.08]	5.50 [1.25]	0.076^†^
TC	4.70 [1.10]	4.80 [1.60]	0.042*	4.20 [1.20]	4.20 [1.43]	0.489
TG	1.40 [1.04]	1.32 [0.93]	0.335	1.19 [0.71]	1.39 [1.03]	0.131
HDL‐C	1.28 [0.40]	1.28 [0.32]	0.909	1.10 [0.30]	1.08 [0.30]	0.969
LDL‐C	2.85 ± 0.90	3.17 ± 0.96	0.011*	2.58 ± 0.94	2.61 ± 0.85	0.809
UA	287.34 ± 70.00	298.96 ± 73.81	0.229	340.00 [83.75]	357.50 [117.50]	0.078^†^
Cr	50.00 [10.00]	54.00 [12.00]	0.048*	69.00 [17.75]	68.00 [16.25]	0.203
MDRD‐eGFR	116.18 ± 21.45	109.68 ± 23.67	0.032*	107.44 ± 24.25	110.77 ± 21.51	0.345
TyG	8.70 [0.97]	8.75 [0.77]	0.144	8.49 [0.77]	8.76 [0.71]	0.050^†^
**Echocardiographic parameters**						
IVS	10.00 [0.88]	10.00 [0.00]	0.264	10.00 [1.00]	10.00 [1.00]	0.873
LVEDD	44.00 [4.00]	44.00 [4.00]	0.412	46.00 [4.00]	46.50 [4.00]	0.014*
LA	34.00 [4.00]	35.00 [3.00]	0.026*	35.61 ± 3.37	36.58 ± 3.83	0.062^†^
LVPW	9.00 [1.00]	10.00 [1.00]	0.027*	10.00 [0.00]	10.00 [0.00]	0.097^†^
RV	30.00 [3.00]	30.00 [3.00]	0.787	31.00 [4.00]	32.00 [3.00]	0.382
RA	31.00 [3.75]	31.00 [4.00]	0.785	32.00 [4.00]	33.00 [3.00]	0.243
LVEF	66.00 [5.75]	66.00 [6.00]	0.644	65.00 [8.00]	65.00 [6.00]	0.693
LV‐EDV	82.00 [15.00]	82.00 [17.00]	0.633	92.50 [18.75]	94.50 [19.50]	0.238
LV‐ESV	27.88 ± 6.00	28.48 ± 6.43	0.474	31.00 [10.50]	33.00 [7.25]	0.183
SV	55.00 [9.75]	53.00 [11.00]	0.905	60.00 [14.00]	61.00 [12.50]	0.300
E/e'	8.57 [3.45]	9.33 [4.62]	0.038*	7.78 [3.27]	8.42 [3.33]	0.384

*Note:* Data are presented as mean ± SD, median [interquartile range], or n (%) *p* values were obtained from independent *t*‐test, Mann–Whitney *U* test, or Chi‐square test as appropriate. *p < 0.05, ^†^ *p* < 0.1. Postmenopausal status is applicable only to female patients.

Abbreviations: BMI, body mass index; Cr, creatinine; DBP, diastolic blood pressure; E/e’, ratio of early diastolic transmitral flow velocity (E) to early diastolic mitral annular tissue velocity (e′); FBG, fasting blood glucose; Hb, hemoglobin; HDL‐C, high‐density lipoprotein cholesterol; HR, heart rate; IVS, interventricular septal thickness; LA, left atrial diameter; LAD‐MFR, left anterior descending myocardial flow reserve; LAD‐rMBF, LAD resting myocardial blood flow; LAD‐sMBF, LAD stress myocardial blood flow; LCX‐MFR, left circumflex myocardial flow reserve; LCX‐rMBF, LCX resting myocardial blood flow; LCX‐sMBF, LCX stress myocardial blood flow; LDL‐C, low‐density lipoprotein cholesterol; LVEDD, left ventricular end‐diastolic diameter; LV‐EDV, left ventricular end‐diastolic volume; LVEF, left ventricular ejection fraction; LV‐ESV, left ventricular end‐systolic volume; LV‐MFR, left ventricular myocardial flow reserve; LVPW, left ventricular posterior wall thickness; LV‐rMBF, left ventricular resting myocardial blood flow; LV‐sMBF, left ventricular stress myocardial blood flow; MDRD‐eGFR, Modification of Diet in Renal Disease estimated glomerular filtration rate; PLT, platelet count; RA, right atrial diameter; RCA‐MFR, right coronary artery myocardial flow reserve; RCA‐rMBF, RCA resting myocardial blood flow; RCA‐sMBF, RCA stress myocardial blood flow; RV, right ventricular diameter; SBP, systolic blood pressure; TC, total cholesterol; TG, triglycerides; TyG, triglyceride‐glucose index; SV, stroke volume; UA, uric acid; WBC, white blood cell count. Units: MFR, unitless (ratio); rMBF and sMBF, mL/(min·g); BMI, kg/m²; SBP and DBP, mmHg; HR, beats/min; WBC and PLT, 10⁹/L; Hb, g/L; FBG, TC, TG, HDL‐C, LDL‐C, mmol/L; TyG, unitless; UA and Cr, μmol/L; MDRD‐eGFR, mL/min/1.73 m²; IVS, LVEDD, LA, LVPW, RV, RA, mm; LV‐EDV, LV‐ESV, SV, mL; LVEF, %; E/e’, unitless.

These candidate variables were subsequently entered into univariate logistic regression analysis in the overall cohort. Significant associations were observed for UA (*p* = 0.030), TyG index (*p* = 0.048), LVEDD (*p* = 0.020), LA (*p* = 0.004), LVPW (*p* = 0.003), and E/e’ ratio (*p* = 0.026). Borderline associations were noted for age (*p* = 0.050), diabetes (*p* = 0.088), LDL‐C (*p* = 0.051), postmenopausal status (*p* = 0.057), FBG (*p* = 0.066), and TC (*p* = 0.095), whereas TG (*p* = 0.498), Cr (*p* = 0.540), and MDRD‐eGFR (*p* = 0.309) were not significant in the overall cohort (Table 2). However, sex‐stratified univariate analysis retained Cr (*p* = 0.020) and MDRD‐eGFR (*p* = 0.035) in females (Table S2). Accordingly, only TG was excluded, resulting in 14 variables for multivariable modeling.

**Table 2:** Logistic regression analysis for abnormal MFR in the overall cohort.

	Univariate		Multivariable (Main effect)		Sex interaction	
	OR (95% CI)	*P* value	OR (95% CI)	*P* value	OR (95% CI)	*P* value
Age	1.023 (1.000–1.046)	0.050^†^	1.001 (0.958–1.046)	0.960	1.032 (0.974–1.094)	0.282
Diabetes	1.486 (0.942–2.344)	0.088^†^	0.838 (0.367–1.911)	0.674	1.985 (0.554–7.108)	0.292
Postmenopausal	2.453 (0.975–6.174)	0.057^†^	1.853 (0.591–5.812)	0.290	—	—
FBG	1.105 (0.993–1.230)	0.066^†^	1.091 (0.885‐1.347)	0.414	0.847 (0.570–1.259)	0.412
TC	1.172 (0.973–1.411)	0.095^†^	—	—	—	—
TG	1.047 (0.916–1.198)	0.498	—	—	—	—
LDL‐C	1.237 (0.999–1.532)	0.051^†^	1.471 (1.072–2.018)	0.017*	0.650 (0.395–1.068)	0.089^†^
UA	1.003 (1.000–1.005)	0.030*	1.001 (0.997‐1.005)	0.729	1.005 (0.998–1.011)	0.145
Cr	1.004 (0.991–1.018)	0.540	—	—	—	—
MDRD‐eGFR	0.995 (0.987–1.004)	0.309	0.991 (0.977–1.005)	0.216	1.024 (1.003–1.046)	0.026*
TyG	1.336 (1.002–1.781)	0.048*	0.954 (0.545–1.671)	0.870	1.437 (0.569–3.627)	0.443
LVEDD	1.084 (1.013–1.160)	0.020*	1.021 (0.897–1.161)	0.757	1.090 (0.906–1.310)	0.360
LA	1.090 (1.028–1.156)	0.004*	1.039 (0.932–1.158)	0.492	0.964 (0.823–1.129)	0.649
LVPW	1.617 (1.179–2.216)	0.003*	1.308 (0.788–2.170)	0.299	1.096 (0.486–2.469)	0.825
E/e'	1.086 (1.010–1.167)	0.026*	1.079 (0.977–1.191)	0.133	0.900 (0.755–1.073)	0.240

*Note: p* values from univariate and multivariable logistic regression analyses, including interaction terms. *p < 0.05, ^†^ *p* < 0.1.

Abbreviations: CI, confidence interval; Cr, creatinine; E/e’, ratio of early diastolic transmitral flow velocity (E) to early diastolic mitral annular tissue velocity (e′); FBG, fasting blood glucose; LA, left atrial diameter; LDL‐C, low‐density lipoprotein cholesterol; LVEDD, left ventricular end‐diastolic diameter; LVPW, left ventricular posterior wall thickness; MDRD‐eGFR, Modification of Diet in Renal Disease estimated glomerular filtration rate; MFR, myocardial flow reserve; OR, odds ratio; TC, total cholesterol; TG, triglycerides; TyG, triglyceride‐glucose index; UA, uric acid. Units: OR, unitless; Cr and UA, μmol/L; MDRD‐eGFR, mL/min/1.73 m²; LA, LVEDD and LVPW, mm; E/e’, unitless; FBG, TC, TG, LDL‐C, mmol/L; TyG, unitless.

Multicollinearity assessment using VIF analysis identified high collinearity within the lipid group (TC VIF = 12.819, LDL‐C VIF = 11.944) and the renal function group (Cr VIF = 10.298, MDRD‐eGFR VIF = 10.784). To enhance model stability, TC and Cr were excluded, retaining LDL‐C and MDRD‐eGFR. Subsequent VIF values were < 2.5 for the remaining 12 variables. A pooled multivariable logistic regression model was then constructed, incorporating sex and the 12 predictors as main effects (age, diabetes, postmenopausal status, FBG, LDL‐C, UA, MDRD‐eGFR, TyG index, LVEDD, LA, LVPW, E/e’ ratio), along with 11 sex interaction terms (excluding postmenopausal status). The overall model was statistically significant (Omnibus *χ*² = 41.535, df = 24, *p* = 0.015), with modest explanatory power (Cox & Snell *R*² = 0.100; Nagelkerke *R*² = 0.134) and good calibration (Hosmer–Lemeshow *χ*² = 6.450, df = 8, *p* = 0.597), achieving a classification accuracy of 63.4%.

**Figure 1:**
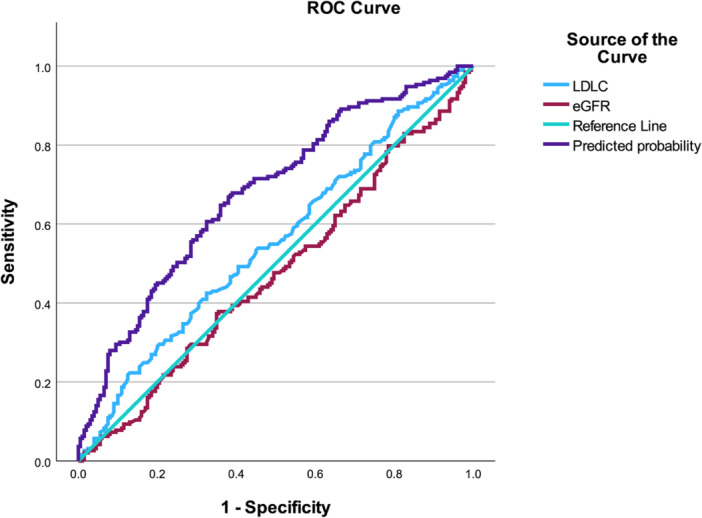
ROC Curve Analysis of Predicted Probability, LDL‐C, and MDRD‐eGFR for Identifying Abnormal MFR in All NOCAD Patients. For AUC values significantly below 0.5, 1 − AUC is reported to aid interpretation, equivalent to reversing the predictor direction.

**Figure 2:**
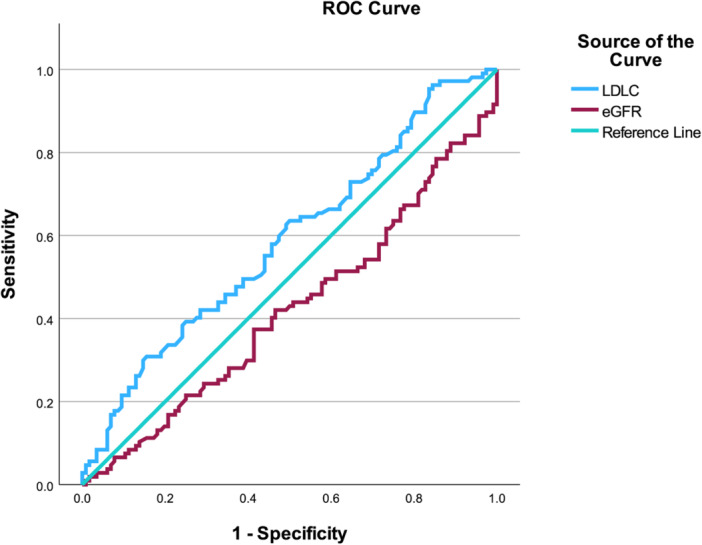
ROC Curve Analysis of LDL‐C and MDRD‐eGFR for Identifying Abnormal MFR in Female NOCAD Patients. For AUC values significantly below 0.5, 1 − AUC is reported to aid interpretation, equivalent to reversing the predictor direction.

**Figure 3:**
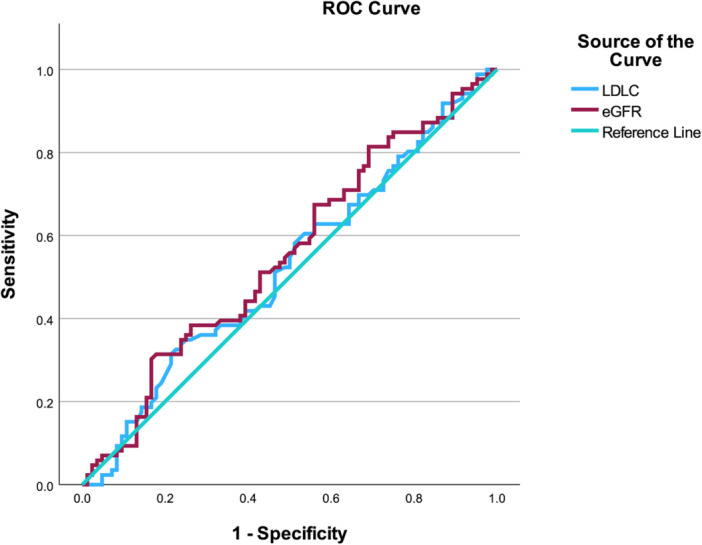
ROC Curve Analysis of LDL‐C and MDRD‐eGFR for Identifying Abnormal MFR in Male NOCAD Patients. For AUC values significantly below 0.5, 1 − AUC is reported to aid interpretation, equivalent to reversing the predictor direction.

In this model (Table 2), LDL‐C was associated with abnormal MFR (OR: 1.471, 95% CI: 1.072–2.018, *p* = 0.017). A significant sex × MDRD‐eGFR interaction was observed (OR: 1.024, 95% CI: 1.003–1.046, *p* = 0.026), with a borderline sex × LDL‐C interaction (OR: 0.650, 95% CI: 0.395–1.068, *p* = 0.089). Sensitivity analyses were conducted to assess the robustness of the primary findings. When a stricter cutoff of MFR < 2.0 was applied, the previously observed sex × MDRD‐eGFR interaction was attenuated and no longer statistically significant, whereas a significant sex × diabetes interaction emerged (Table S3). In additional analyses modeling MFR as a continuous outcome, continuous variables involved in interaction terms were mean‐centered prior to model construction to minimize multicollinearity. The sex × MDRD‐eGFR interaction remained directionally consistent (*β* = −0.007, *p* = 0.088), while the sex × diabetes interaction remained statistically significant (*β* = −0.589, *p* = 0.027) (Table S4). These findings were directionally aligned with the primary interaction analysis but suggest sensitivity to cutoff and model specification. ROC analysis further evaluated model performance, with the predicted probability yielding an AUC of 0.680 (95% CI: 0.627–0.732), *p* < 0.001 in the overall cohort, outperforming LDL‐C (AUC: 0.554, 95% CI: 0.497–0.611), *p* = 0.064 and MDRD‐eGFR (AUC: 0.476, 95% CI: 0.419–0.533), *p* = 0.416. In females, LDL‐C showed an AUC of 0.586 (95% CI: 0.511–0.661), *p* = 0.024, while MDRD‐eGFR yielded an AUC of 0.420 (95% CI: 0.345–0.495), *p* = 0.036. Because this value was < 0.5, the reversed AUC (1‐AUC = 0.580) is presented to reflect the inverse discrimination direction. In males, neither variable demonstrated discriminatory ability (LDL‐C AUC: 0.525, 95% CI: 0.438–0.612), *p* = 0.569; MDRD‐eGFR AUC: 0.552 (95% CI: 0.466–0.639), *p* = 0.237 (Figures 1, 2, 3).

### 3.3 Sex‐Specific Phenotypes and Correlations in Patients With Abnormal MFR

**Figure 4:**
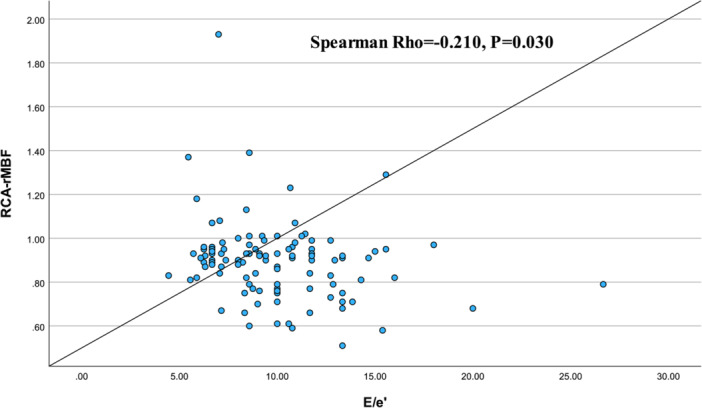
Scatter Plot and Correlation Analysis of RCA‐rMBF and E/e’ in Female Patients with Abnormal MFR.

Among the 193 patients with abnormal MFR (< 2.5), significant differences were observed between males (*n* = 86) and females (*n* = 107) (Table 3). Females exhibited higher resting myocardial blood flow (rMBF) in the left circumflex (LCX) territory (*p* = 0.012), right coronary artery (RCA) territory (*p* = 0.020), and an elevated E/e’ ratio (*p* = 0.011). Trends were noted for overall left ventricular rMBF (LV‐rMBF, *p* = 0.072) and age (*p* = 0.072). Additionally, correlation analysis in females with abnormal MFR revealed a significant negative association between RCA‐rMBF and E/e’ ratio (Spearman Rho = −0.210, *p* = 0.030) (Figure 4), with no significant correlations for LV‐rMBF or LCX‐rMBF with E/e’ (*p* > 0.05).

**Table 3:** Clinical phenotype differences between male and female patients with abnormal MFR.

	Male with abnormal MFR (*n* = 86)	Female with abnormal MFR (*n* = 107)	*P* value (*t*‐test)	Interaction *P* value
**CZT‐SPECT parameters**				
LV‐MFR	1.97 [0.59]	1.86 [0.68]	0.425	—
LAD‐MFR	1.92 ± 0.44	1.91 ± 0.47	0.955	—
LCX‐MFR	1.77 ± 0.45	1.69 ± 0.45	0.215	—
RCA‐MFR	1.97 ± 0.47	1.91 ± 0.48	0.387	—
LV‐rMBF	0.88 [0.14]	0.89 [0.12]	0.072^†^	—
LV‐sMBF	1.56 ± 0.43	1.61 ± 0.41	0.376	—
LAD‐rMBF	0.91 [0.13]	0.90 [0.15]	0.892	—
LAD‐sMBF	1.65 [0.64]	1.59 [0.50]	0.747	—
LCX‐rMBF	0.82 [0.22]	0.85 [0.15]	0.012*	—
LCX‐sMBF	1.39 ± 0.44	1.44 ± 0.45	0.372	—
RCA‐rMBF	0.88 [0.19]	0.91 [0.16]	0.020*	—
RCA‐sMBF	1.62 [0.85]	1.59 [0.72]	0.484	—
**Baseline characteristics**				
Age	61.50 [15.25]	63.00 [11.00]	0.072^†^	0.282
BMI	26.05 [4.26]	24.97 [4.35]	0.111	—
Hypertension	47 (54.7%)	61 (57.0%)	0.743	—
Diabetes	25 (29.1%)	32 (29.9%)	0.899	0.292
Current smoking	36 (41.9%)	14 (13.1%)	< 0.001*	—
**Vital signs**				
Resting SBP	136.00 [21.00]	133.00 [19.00]	0.672	—
Resting DBP	80.72 ± 10.08	78.59 ± 10.06	0.145	—
Resting HR	70.50 [14.00]	72.00 [12.00]	0.423	—
**Laboratory values**				
WBC	5.70 [1.73]	5.30 [1.80]	0.036*	—
Hb	150.65 ± 14.08	133.03 ± 9.60	< 0.001*	—
PLT	210.00 [76.50]	241.00 [71.00]	< 0.001*	—
FBG	5.50 [1.25]	5.40 [1.70]	0.791	0.412
TC	4.20 [1.43]	4.80 [1.60]	<0.001*	—
TG	1.39 [1.03]	1.32 [0.93]	0.520	—
HDL‐C	1.08 [0.30]	1.28 [0.32]	< 0.001*	—
LDL‐C	2.61 ± 0.85	3.17 ± 0.96	< 0.001*	0.089^†^
UA	368.22 ± 86.79	298.96 ± 73.81	< 0.001*	0.145
Cr	68.00 [16.25]	54.00 [12.00]	< 0.001*	—
MDRD‐eGFR	110.77 ± 21.51	109.68 ± 23.67	0.741	0.026*
TyG	8.75 [0.77]	8.76 [0.71]	0.341	0.443
**Echocardiographic parameters**				
IVS	10.00 [1.00]	10.00 [0.00]	< 0.001*	—
LVEDD	46.50 [4.00]	44.00 [4.00]	< 0.001*	0.360
LA	37.00 [5.00]	35.00 [3.00]	0.005*	0.649
LVPW	10.00 [0.00]	10.00 [1.00]	< 0.001*	0.825
RV	32.00 [3.00]	30.00 [3.00]	< 0.001*	—
RA	33.00 [3.00]	31.00 [4.00]	< 0.001*	—
LVEF	65.00 [6.00]	66.00 [6.00]	0.223	—
LV‐EDV	94.50 [19.50]	82.00 [17.00]	< 0.001*	—
LV‐ESV	33.29 ± 6.44	28.48 ± 6.43	< 0.001*	—
SV	61.00 [12.50]	53.00 [11.00]	< 0.001*	—
E/e'	8.42 [3.33]	9.33 [4.62]	0.011*	0.240

*Note:* Data are presented as mean ± SD, median [interquartile range], or n (%). *p* values were obtained from independent *t*‐test, Mann–Whitney *U* test, or Chi‐square test as appropriate. Interaction p values were obtained from multivariable logistic regression models including sex‐by‐variable interaction terms. *p < 0.05, ^†^ *p* < 0.1.

Abbreviations: BMI, body mass index; Cr, creatinine; DBP, diastolic blood pressure; E/e', ratio of early diastolic transmitral flow velocity (E) to early diastolic mitral annular tissue velocity (e'); FBG, fasting blood glucose; Hb, hemoglobin; HDL‐C, high‐density lipoprotein cholesterol; HR, heart rate; IVS, interventricular septal thickness; LA, left atrial diameter; LAD‐MFR, left anterior descending myocardial flow reserve; LAD‐rMBF, LAD resting myocardial blood flow; LAD‐sMBF, LAD stress myocardial blood flow; LCX‐MFR, left circumflex myocardial flow reserve; LCX‐rMBF, LCX resting myocardial blood flow; LCX‐sMBF, LCX stress myocardial blood flow; LDL‐C, low‐density lipoprotein cholesterol; LVEDD, left ventricular end‐diastolic diameter; LV‐EDV, left ventricular end‐diastolic volume; LVEF, left ventricular ejection fraction; LV‐ESV, left ventricular end‐systolic volume; LV‐MFR, left ventricular myocardial flow reserve; LVPW, left ventricular posterior wall thickness; LV‐rMBF, left ventricular resting myocardial blood flow; LV‐sMBF, left ventricular stress myocardial blood flow; MDRD‐eGFR, modification of diet in renal disease estimated glomerular filtration rate; PLT, platelet count; RA, right atrial diameter; RCA‐MFR, right coronary artery myocardial flow reserve; RCA‐rMBF, RCA resting myocardial blood flow; RCA‐sMBF, RCA stress myocardial blood flow; RV, right ventricular diameter; SBP, systolic blood pressure; TC, total cholesterol; TG, triglycerides; TyG, triglyceride–glucose index; SV, stroke volume; UA, uric acid; WBC, white blood cell count. Units: MFR, unitless (ratio); rMBF and sMBF, mL/(min·g); BMI, kg/m^2^; SBP and DBP, mmHg; HR, beats/min; WBC and PLT, 10^9^/L; Hb, g/L; FBG, TC, TG, HDL‐C, LDL‐C, mmol/L; TyG, unitless; UA and Cr, μmol/L; MDRD‐eGFR, mL/min/1.73 m^2^; IVS, LVEDD, LA, LVPW, RV, RA, mm; LV‐EDV, LV‐ESV, SV, mL; LVEF, %; E/e', unitless.

## 4 Discussion

This real‐world CZT‐SPECT study in NOCAD patients identified LDL‐C as associated with abnormal MFR in the pooled model, with a trend toward sex‐modified LDL‐C effects. A statistically significant sex × MDRD‐eGFR interaction was observed, suggesting that the association between eGFR decline and MFR impairment may differ by sex, with a potentially stronger relationship in males; however, this finding should be interpreted as exploratory and hypothesis‐generating. ROC analyses indicated moderate discriminatory performance of the multivariable model, with female‐specific discrimination for LDL‐C. Additionally, phenotypic patterns among females with abnormal MFR—including elevated territorial rMBF and E/e’ ratios and a negative RCA‐rMBF–E/e’ correlation—may reflect sex‐related cardiorenal‐microvascular interactions that warrant further investigation.

The observed interaction between sex and MDRD‐eGFR may suggest that declining renal function could be differentially associated with microvascular impairment in men, potentially through mechanisms such as endothelial susceptibility or vascular stiffness, which may contribute to microvascular rarefaction and fibrosis [21, 22]. Clinically, these findings raise the possibility that modest eGFR reductions in males could signal heightened CMVD vulnerability; however, prospective validation is required before clinical implementation [23, 24]. Mechanistically, LDL‐C's established role in microvascular plaque deposition aligns with prior evidence [25], and the observed sex × LDL‐C trend (*p* = 0.089) may indicate potential sex‐related variability in lipid‐associated microvascular dysfunction, possibly influenced by estrogen‐modulated oxidative pathways [26, 27]. Because baseline lipid‐lowering therapy information was unavailable, LDL‐C levels should be interpreted as measured clinical values at the time of testing, which may reflect a combination of underlying lipid exposure and treatment effects. Existing MFR predictors include diabetes and hypertension, with sex differences in diabetic effects previously reported [28, 29]; however, our findings suggest a potential renal‐lipid interaction that may differ by sex in NOCAD, which warrants further confirmation. ROC outcomes showed a model AUC of 0.680, with female LDL‐C AUC of 0.586 indicating modest discrimination [30]. These results may inform future investigations of sex‐informed preventive strategies but should be interpreted cautiously.

Phenotypes in abnormal MFR, with female elevations in LCX‐rMBF, RCA‐rMBF, and E/e’, fused with the sex×MDRD‐eGFR interaction, may imply renal‐modulated perfusion shifts aggravating diastolic burden, possibly reflecting compensatory microvascular adaptations in the context of impaired relaxation—a pattern borrowing from original observations of regional microvascular adaptations [31, 32]. The RCA‐rMBF–E/e’ correlation (Rho = −0.210, *p* = 0.030) further suggests regional flow may be associated with attenuation of diastolic strain, mechanistically linked to nitric oxide dysregulation in estrogen‐deficient microvasculature, providing clinical cues for territory‐targeted vasodilators and insights into female CMVD resilience.

The 49.1% abnormal MFR rate echoes CMVD prevalence in NOCAD (40–60%), affirming its epidemiological scope and the imperative for functional testing like CZT‐SPECT to refine risk stratification beyond anatomical imaging, particularly in symptomatic patients [2, 33, 34]. CZT‐SPECT's merits—cost‐efficiency, low radiation, and MFR precision rivaling PET—bolster its promise for routine CMVD screening, enabling early intervention in diverse settings. The MFR cutoff of 2.5 was selected to prioritize sensitivity for early CMVD detection, as validated by literature demonstrating high sensitivity (86%) and specificity (73%) for identifying microvascular dysfunction in non‐invasive imaging, while the more prevalent 2.0 cutoff emphasizes prognostic utility in predicting cardiovascular events [35, 36]. Our focus on diagnostic rather than prognostic aspects justified including patients with 2.0 ≤ LV‐MFR < 2.5, categorized as borderline abnormal per institutional nuclear medicine protocols. Clinical observations indicate these patients often exhibit typical symptoms and respond favorably to microvascular therapies, supporting their inclusion as abnormal to enable earlier high‐risk identification, though larger studies are needed to validate this threshold's clinical impact.

Limitations of this study include its retrospective design and single‐center setting, which may limit generalizability. In addition, baseline lipid‐lowering therapy was not reliably documented in the medical records and therefore could not be adjusted for in the analysis; this may have influenced the observed association between LDL‐C and MFR. Interaction signals were sensitive to the choice of MFR cutoff, and findings from continuous modeling should be interpreted as exploratory and hypothesis‐generating, particularly given the number of interaction terms tested. In addition, no internal validation procedures (such as bootstrap resampling or cross‐validation) were performed for the multivariable models, which may limit the robustness of the findings. Future efforts should pursue multi‐center validation of sex interactions, longitudinal outcome assessment, and prospective studies to clarify renal‐lipid mechanisms in sex‐specific CMVD.

## 5 Conclusions

This CZT‐SPECT study in NOCAD suggests potential sex‐modified associations of renal and lipid parameters with abnormal MFR. The observed interaction patterns and sex‐specific phenotypes provide preliminary insights into cardiorenal‐microvascular relationships in CMVD, but should be considered exploratory and hypothesis‐generating and require further validation in independent cohorts.

## Author Contributions

**Ya‐Jie Wang:** conceptualization, data curation, formal analysis, investigation, methodology, validation, visualization, writing – original draft. **Jian‐Ming Li:** investigation, resources, software, validation, writing – review and editing. **Xue Wu:** writing – review and editing. **Tong Liu:** funding acquisition, project administration, supervision, validation, writing – review and editing. **Kang‐Yin Chen:** funding acquisition, project administration, supervision, validation, writing – review and editing. All authors read and approved the final manuscript.

## Ethics Statement

This retrospective study was approved by the Ethics Committee for Research Projects of TEDA International Cardiovascular Hospital (Approval No. [2022]‐0720‐2). The study utilized existing medical records of patients previously admitted to TEDA International Cardiovascular Hospital.

## Consent

Patients had provided general written consent for the use of their data for research purposes through the hospital's admission notification form (completed within 24 h of admission). The Ethics Committee waived the requirement for additional informed consent due to the retrospective nature of the study.

## Conflicts of Interest

The authors declare no conflicts of interest.

## Supporting information

Supporting File 1

Supporting File 2

Supporting File 3

Supporting File 4

Supporting File 5

## Data Availability

The datasets used and/or analyzed during the current study are available from the corresponding author upon reasonable request.
