# Supplementary figures and images for: Sex‐Specific Associations With Abnormal Myocardial Flow Reserve in Non‐Obstructive Coronary Artery Disease: Insights From a Real‐World Cadmium‐Zinc‐Telluride SPECT Study
Source: Clin Cardiol. 2026 Apr 23;49(4):e70294. doi: 10.1002/clc.70294 (PMC13104727; doi:10.1002/clc.70294)

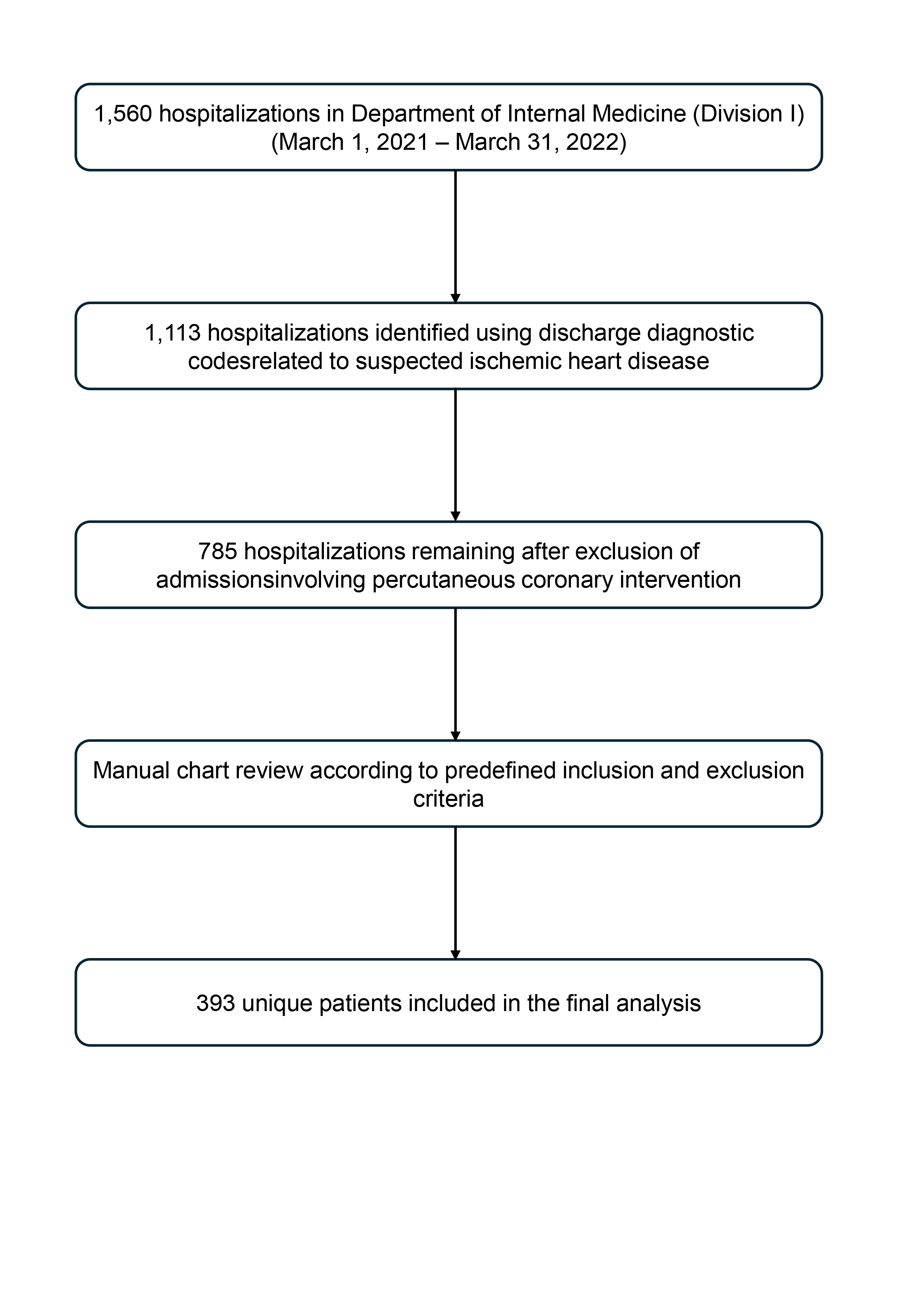

Supplement: Supplementary file 1 — Supporting File 1 [file CLC-49-e70294-s002.tif]
