# Supplementary material for: Sex‐Specific Associations With Abnormal Myocardial Flow Reserve in Non‐Obstructive Coronary Artery Disease: Insights From a Real‐World Cadmium‐Zinc‐Telluride SPECT Study
Source: Clin Cardiol. 2026 Apr 23;49(4):e70294. doi: 10.1002/clc.70294 (PMC13104727; doi:10.1002/clc.70294)
Supplement: Supplementary file 2 — Supporting File 2 [file CLC-49-e70294-s001.docx]

Supplementary Table 1. Comparison of Patient Characteristics in the Overall Cohort by MFR Status

|  | **Normal MFR group (n=200)** | **Abnormal MFR group (n=193)** | **P value** |
| --- | --- | --- | --- |
| **CZT-SPECT Parameters** | |  |  |
| LV-MFR | 3.23 [0.87] | 1.93 [0.63] | <0.001^*^ |
| LAD-MFR | 3.23 [0.91] | 1.95 [0.66] | <0.001^*^ |
| LCX-MFR | 3.02 [0.84] | 1.76 [0.65] | <0.001^*^ |
| RCA-MFR | 3.54 ± 0.70 | 1.94 ± 0.48 | <0.001^*^ |
| LV-rMBF | 0.90 [0.15] | 0.89 [0.14] | 0.572 |
| LV-sMBF | 2.83 ± 0.72 | 1.59 ± 0.42 | <0.001^*^ |
| LAD-rMBF | 0.91 [0.18] | 0.90 [0.14] | 0.930 |
| LAD-sMBF | 2.74 [1.12] | 1.61 [0.55] | <0.001^*^ |
| LCX-rMBF | 0.84 [0.17] | 0.84 [0.15] | 0.627 |
| LCX-sMBF | 2.47 [0.88] | 1.38 [0.64] | <0.001^*^ |
| RCA-rMBF | 0.90 [0.19] | 0.89 [0.16] | 0.811 |
| RCA-sMBF | 3.06 [1.51] | 1.59 [0.73] | <0.001^*^ |
| **Baseline Characteristics** | |  |  |
| Age | 59.97 ± 9.21 | 61.75 ± 8.67 | 0.049^*^ |
| BMI | 25.43 [4.77] | 25.39 [4.43] | 0.844 |
| Hypertension | 112 (56.0%) | 108 (56.0%) | 0.993 |
| Diabetes | 44 (22.0%) | 57 (29.5%) | 0.088^†^ |
| Current smoking | 39 (19.5%) | 50 (25.9%) | 0.129 |
| Postmenopausal | 99 (85.3%) | 100 (93.5%) | 0.051^†^ |
| **Vital Signs** | |  |  |
| Resting SBP | 133.44 ± 14.78 | 134.36 ± 15.72 | 0.551 |
| Resting DBP | 79.83 ± 10.83 | 79.54 ± 10.10 | 0.787 |
| Resting HR | 73.00 [16.00] | 71.00 [12.50] | 0.177 |
| **Laboratory Values** | |  |  |
| WBC | 5.60 [1.90] | 5.50 [1.95] | 0.746 |
| Hb | 138.50 [22.00] | 139.00 [20.50] | 0.269 |
| PLT | 229.50 [75.75] | 228.00 [69.50] | 0.588 |
| FBG | 5.30 [1.30] | 5.50 [1.60] | 0.042^*^ |
| TC | 4.50 [1.20] | 4.70 [1.40] | 0.085^†^ |
| TG | 1.27 [0.96] | 1.35 [0.98] | 0.093^†^ |
| HDL-C | 1.19 [0.37] | 1.20 [0.35] | 0.873 |
| LDL-C | 2.73 ± 0.92 | 2.92 ± 0.95 | 0.050^†^ |
| UA | 311.63 ± 77.22 | 329.82 ± 86.80 | 0.029^*^ |
| Cr | 57.00 [19.00] | 59.00 [17.50] | 0.252 |
| MDRD-eGFR | 112.51 ± 23.02 | 110.16 ± 22.68 | 0.309 |
| TyG | 8.58 [0.87] | 8.76 [0.78] | 0.020^*^ |
| **Echocardiographic Parameters** | |  |  |
| IVS | 10.00 [0.50] | 10.00 [1.00] | 0.210 |
| LVEDD | 45.00 [4.00] | 46.00 [4.00] | 0.022^*^ |
| LA | 35.00 [5.00] | 36.00 [4.00] | 0.004^*^ |
| LVPW | 10.00 [1.00] | 10.00 [1.00] | 0.004^*^ |
| RV | 30.00 [3.75] | 30.00 [3.00] | 0.329 |
| RA | 31.00 [3.00] | 32.00 [4.00] | 0.431 |
| LVEF | 65.00 [6.00] | 66.00 [7.00] | 0.818 |
| LV-EDV | 85.00 [19.00] | 88.00 [21.00] | 0.164 |
| LV-ESV | 29.00 [9.00] | 31.00 [10.50] | 0.101 |
| SV | 56.00 [12.00] | 57.00 [14.00] | 0.404 |
| E/e’ | 8.33 [3.00] | 8.89 [3.85] | 0.045^*^ |

*Note:* Data are presented as mean ± SD, median [interquartile range], or n (%). *p* values were obtained from independent t-test, Mann–Whitney U test, or Chi-square test as appropriate. ^*^*p* < 0.05, ^†^*p* < 0.1. Postmenopausal status is applicable only to female patients.

Abbreviations: BMI, body mass index; Cr, creatinine; DBP, diastolic blood pressure; E/e’, ratio of early diastolic transmitral flow velocity (E) to early diastolic mitral annular tissue velocity (e'); FBG, fasting blood glucose; Hb, hemoglobin; HDL-C, high-density lipoprotein cholesterol; HR, heart rate; IVS, interventricular septal thickness; LA, left atrial diameter; LAD-MFR, left anterior descending myocardial flow reserve; LAD-rMBF, LAD resting myocardial blood flow; LAD-sMBF, LAD stress myocardial blood flow; LCX-MFR, left circumflex myocardial flow reserve; LCX-rMBF, LCX resting myocardial blood flow; LCX-sMBF, LCX stress myocardial blood flow; LDL-C, low-density lipoprotein cholesterol; LVEDD, left ventricular end-diastolic diameter; LV-EDV, left ventricular end-diastolic volume; LVEF, left ventricular ejection fraction; LV-ESV, left ventricular end-systolic volume; LV-MFR, left ventricular myocardial flow reserve; LVPW, left ventricular posterior wall thickness; LV-rMBF, left ventricular resting myocardial blood flow; LV-sMBF, left ventricular stress myocardial blood flow; MDRD-eGFR, Modification of Diet in Renal Disease estimated glomerular filtration rate; PLT, platelet count; RA, right atrial diameter; RCA-MFR, right coronary artery myocardial flow reserve; RCA-rMBF, RCA resting myocardial blood flow; RCA-sMBF, RCA stress myocardial blood flow; RV, right ventricular diameter; SBP, systolic blood pressure; TC, total cholesterol; TG, triglycerides; TyG, triglyceride–glucose index; SV, stroke volume; UA, uric acid; WBC, white blood cell count. Units: MFR, unitless (ratio); rMBF and sMBF, mL/(min·g); BMI, kg/m²; SBP and DBP, mmHg; HR, beats/min; WBC and PLT, 10⁹/L; Hb, g/L; FBG, TC, TG, HDL-C, LDL-C, mmol/L; TyG, unitless; UA and Cr, μmol/L; MDRD-eGFR, mL/min/1.73 m²; IVS, LVEDD, LA, LVPW, RV, RA, mm; LV-EDV, LV-ESV, SV, mL; LVEF, %; E/e’, unitless.
