# Supplementary material for: Sex‐Specific Associations With Abnormal Myocardial Flow Reserve in Non‐Obstructive Coronary Artery Disease: Insights From a Real‐World Cadmium‐Zinc‐Telluride SPECT Study
Source: Clin Cardiol. 2026 Apr 23;49(4):e70294. doi: 10.1002/clc.70294 (PMC13104727; doi:10.1002/clc.70294)
Supplement: Supplementary file 3 — Supporting File 3 [file CLC-49-e70294-s005.docx]

Supplementary Table 2. Sex-Stratified Univariate Logistic Regression for Abnormal MFR Associations

|  |  | **OR (95% CI)** | **P value** |
| --- | --- | --- | --- |
| **Female NOCAD patients** | | |  |
|  | **Age** | 1.035 (1.001–1.069) | 0.041^*^ |
|  | **Postmenopausal** | 2.453 (0.975–6.174) | 0.057^†^ |
|  | **TC** | 1.312 (1.014–1.699) | 0.039^*^ |
|  | **LDL-C** | 1.456 (1.086–1.951) | 0.012^*^ |
|  | **Cr** | 1.037 (1.006–1.070) | 0.020^*^ |
|  | **MDRD-eGFR** | 0.987 (0.975–0.999) | 0.035^*^ |
|  | **LA** | 1.090 (1.007–1.180) | 0.032^*^ |
|  | **LVPW** | 1.627 (1.089–2.432) | 0.018^*^ |
|  | **E/e’** | 1.105 (1.008–1.211) | 0.034^*^ |
| **Male NOCAD patients** | | |  |
|  | **Diabetes** | 1.885 (0.911–3.900) | 0.087^†^ |
|  | **FBG** | 1.111 (0.921–1.340) | 0.271 |
|  | **UA** | 1.004 (1.000–1.007) | 0.067^†^ |
|  | **TyG** | 1.351 (0.888–2.055) | 0.160 |
|  | **LVEDD** | 1.130 (1.016–1.258) | 0.025^*^ |
|  | **LA** | 1.091 (0.995–1.196) | 0.064^†^ |
|  | **LVPW** | 1.651 (0.942–2.894) | 0.080^†^ |

*Note: p* values were obtained from univariate logistic regression analysis. ^*^*p* < 0.05, ^†^*p* < 0.1.

Abbreviations: CI, confidence interval; Cr, creatinine; E/e’, ratio of early diastolic transmitral flow velocity (E) to early diastolic mitral annular tissue velocity (e'); FBG, fasting blood glucose; LA, left atrial diameter; LDL-C, low-density lipoprotein cholesterol; LVPW, left ventricular posterior wall thickness; LVEDD, left ventricular end-diastolic diameter; MDRD-eGFR, Modification of Diet in Renal Disease estimated glomerular filtration rate; MFR, myocardial flow reserve; NOCAD, non-obstructive coronary artery disease; OR, odds ratio; TC, total cholesterol; TyG, triglyceride-glucose index; UA, uric acid. Units: Cr and UA, μmol/L; LA, LVEDD, LVPW, mm; E/e’, unitless; FBG, LDL-C, TC, mmol/L; MDRD-eGFR, mL/min/1.73 m²; TyG, unitless.
