# Supplementary material for: Sex‐Specific Associations With Abnormal Myocardial Flow Reserve in Non‐Obstructive Coronary Artery Disease: Insights From a Real‐World Cadmium‐Zinc‐Telluride SPECT Study
Source: Clin Cardiol. 2026 Apr 23;49(4):e70294. doi: 10.1002/clc.70294 (PMC13104727; doi:10.1002/clc.70294)
Supplement: Supplementary file 4 — Supporting File 4 [file CLC-49-e70294-s003.docx]

Supplementary Table 3. Multivariable Logistic Regression Analysis Using MFR <2.0 as the Definition of Abnormality in the Overall Cohort

| **Variable** | **OR (95% CI)** | **P value** |
| --- | --- | --- |
| LDL-C | 1.120 (0.806–1.558) | 0.499 |
| MDRD-eGFR | 0.998 (0.983–1.013) | 0.793 |
| Diabetes | 0.413 (0.157–1.089) | 0.074^†^ |
| Sex × LDL-C | 0.943 (0.550–1.616) | 0.830 |
| Sex × MDRD-eGFR | 1.006 (0.984–1.029) | 0.577 |
| Sex × Diabetes | 6.714 (1.664–27.084) | 0.007^*^ |

*Note:* *p* values were derived from the multivariable logistic regression model, including interaction terms. ^*^*p* < 0.05, ^†^*p* < 0.10 (two-sided). The multivariable model included the same set of covariates and interaction terms as in the primary analysis.

Abbreviations: MFR, myocardial flow reserve; OR, odds ratio; CI, confidence interval; LDL-C, low-density lipoprotein cholesterol; MDRD-eGFR, Modification of Diet in Renal Disease estimated glomerular filtration rate.
