# Supplementary material for: Sex‐Specific Associations With Abnormal Myocardial Flow Reserve in Non‐Obstructive Coronary Artery Disease: Insights From a Real‐World Cadmium‐Zinc‐Telluride SPECT Study
Source: Clin Cardiol. 2026 Apr 23;49(4):e70294. doi: 10.1002/clc.70294 (PMC13104727; doi:10.1002/clc.70294)
Supplement: Supplementary file 5 — Supporting File 5 [file CLC-49-e70294-s004.docx]

Supplementary Table 4. Multivariable Linear Regression Analysis with MFR as a Continuous Outcome

| **Variable** | **β (95% CI)** | **P value** |
| --- | --- | --- |
| LDL-C_c | -0.074 (-0.201 to 0.054) | 0.256 |
| MDRD-eGFR_c | 0.003 (-0.003 to 0.008) | 0.379 |
| Diabetes | 0.196 (-0.138 to 0.530) | 0.248 |
| Sex × LDL-C_c | 0.081 (-0.121 to 0.282) | 0.431 |
| Sex × MDRD-eGFR_c | -0.007 (-0.016 to 0.001) | 0.088^†^ |
| Sex × Diabetes | -0.589 (-1.110 to -0.067) | 0.027^*^ |

*Note:* Continuous variables involved in interaction terms were mean-centered prior to interaction construction to minimize multicollinearity. β denotes the unstandardized regression coefficient from the multivariable linear regression model including interaction terms. ^*^*p* < 0.05, ^†^*p* < 0.10 (two-sided).

Abbreviations: MFR, myocardial flow reserve; CI, confidence interval; LDL-C, low-density lipoprotein cholesterol; MDRD-eGFR, Modification of Diet in Renal Disease estimated glomerular filtration rate.
